# An Imputation Approach for Oligonucleotide Microarrays

**DOI:** 10.1371/journal.pone.0058677

**Published:** 2013-03-07

**Authors:** Ming Li, Yalu Wen, Qing Lu, Wenjiang J. Fu

**Affiliations:** 1 Division of Biostatistics, Department of Pediatrics, University of Arkansas for Medical Sciences, Little Rock, Arkansas, United States of America; 2 Department of Epidemiology and Biostatistics, Michigan State University, East Lansing, Michigan, United States of America; Deutsches Krebsforschungszentrum, Germany

## Abstract

Oligonucleotide microarrays are commonly adopted for detecting and qualifying the abundance of molecules in biological samples. Analysis of microarray data starts with recording and interpreting hybridization signals from CEL images. However, many CEL images may be blemished by noises from various sources, observed as “bright spots”, “dark clouds”, and “shadowy circles”, etc. It is crucial that these image defects are correctly identified and properly processed. Existing approaches mainly focus on detecting defect areas and removing affected intensities. In this article, we propose to use a mixed effect model for imputing the affected intensities. The proposed imputation procedure is a single-array-based approach which does not require any biological replicate or between-array normalization. We further examine its performance by using Affymetrix high-density SNP arrays. The results show that this imputation procedure significantly reduces genotyping error rates. We also discuss the necessary adjustments for its potential extension to other oligonucleotide microarrays, such as gene expression profiling. The R source code for the implementation of approach is freely available upon request.

## Introduction

Oligonucleotide microarrays have been commonly adopted in various biomedical researches, such as gene expression profiling, single nucleotide polymorphism (SNP) genotyping, and copy number estimation, etc [1]-[3]. Typically, a microarray is attached with millions of short immobilized nucleic acid sequences, known as probes, which are designed as sequences complementary to the nucleic acid molecules in biological samples, known as targets. The targets are usually labeled with fluorescent dyes and their abundance can be qualified by measuring fluorescent intensities yielded from their hybridization with the probes. The intensities are further stored in a CEL file, which becomes the raw data of a microarray experiment (See [4], [5] for detail of cDNA chips). This technology is able to produce a large amount of data for thousands of genes or millions of SNPs simultaneously. However, the quality of a microarray may be affected by noises from various sources during the experiment. A series of pre-processing procedures are required before any subsequent analyses can be conducted, including image processing, background adjustment and data normalization [6].

All fluorescence images may have spatial defects to some extent, due to dust and debris, glass flaws, uneven distribution of fluids or surface coatings, etc [7]. Image processing is usually the first step to ensure the validity of downstream analyses. The intensities from a defect area will distort the scaling [8], which may further affect multiple samples through between array normalizations. The impact of image defects have been investigated by using a number of summarization packages available in bio-conductor, such as MAS5, RMA and GCRMA [9]–[11]. Suárez-Fariñas *et al.* studied the impact of blemished images on gene expression microarrays by directly applying MAS5 and GCRMA [7]. They concluded that a defect area of ∼0.2% of a chip would artificially change the expression by two folds for 20 genes in MAS5, and for 3 genes in GCRMA. Reimers *et al.* conducted a similar comparison by using packages MAS5 and RMA [12]. They found that RMA was less affected than MAS5 when the blemished area was small, but its performance got worse with larger blemished regions [13].

To ensure the image quality, researchers were always recommended to visually examine all CEL images [14], [15]. However, some defects cannot be easily recognized by naked eyes due to a large dynamic range of probe intensities [13]. In the past few years, several approaches have been proposed for detecting and removing the defect areas automatically. These approaches covered a wide variety of image defects. For example, Suárez-Fariñas *et al.* developed an approach, referred to as “harshlight”, which was able to detect and mask three types of defects: localized blemishes affecting a few probes, diffuse defects affecting larger areas, and extended defects which may invalidate an entire chip [7]. Upton *et al.* used replications for identifying the abnormal spatial structures, referred to as “blob”, “lines”, “rectangular enhancement” and “coffee rings” [16]. Song *et al.* proposed a software package, referred to as Microarray Blob Remover (MBR), which allowed visualization, detection and removal of various ‘blob’ like defects [17].

These approaches have showed great promise for image processing. However, the existing approaches commonly masked affected intensities with missing. Previous studies indicated that correction of the affected intensities could improve the reliability of data, and thus improve the reproducibility of the results [7]. However, relatively few strategies are available to impute the affected intensities. Upton *et al.* and Arteaga-Salas discussed possible correction for the affected intensities in some samples [16], [18]. However, the correction relied on biological replicates which were not always available. Imputation approaches based on intensities from the same array are still in great need, especially when working with rare or expensive arrays [13].

In this article, we propose an imputation approach for the intensities from defect areas. The proposed approach is single-array-based, which does not require any biological replicate. It models the cross-hybridization between probes and targets, and imputes the intensities by the binding affinities between them. In the following, we first explain our approach and further examine its performance with Affymetrix high-density SNP arrays. The performance of imputation is evaluated by genotyping accuracies.

## Methods

Among current platforms, Affymetrix DNA microarrays have been commonly used for gene expression profiling, SNP genotyping and copy number estimation at a relatively high resolution with low-cost. In this article, we introduce our approach by using Affymetrix high density SNP arrays. We used SNP arrays as an example, because the performance of imputation can be easily examined by genotyping accuracies.

The Affymetric SNP array is a major platform that was used in the international Haplotype Map (HapMap) projects [19]. The genotypes of HapMap samples are commonly viewed as a gold-standard for the assessment of many genotyping approaches. However, we still found a number of defect areas in a few HapMap arrays, showing bright spots in the CEL images. Though the defect areas are relatively small in HapMap samples, a large number of SNPs could be affected. The intensities from these defect areas are not biologically meaningful and may lead to large genotyping errors and bias in copy number estimation. Most of current genotyping approaches used multi-array training [20]–[24], and relied on the between array normalization to take care of the image defects [25]. It is rarely evaluated how the genotyping results are affected by these defect areas.

### The design of Affymetrix SNP arrays

**Figure 1:**
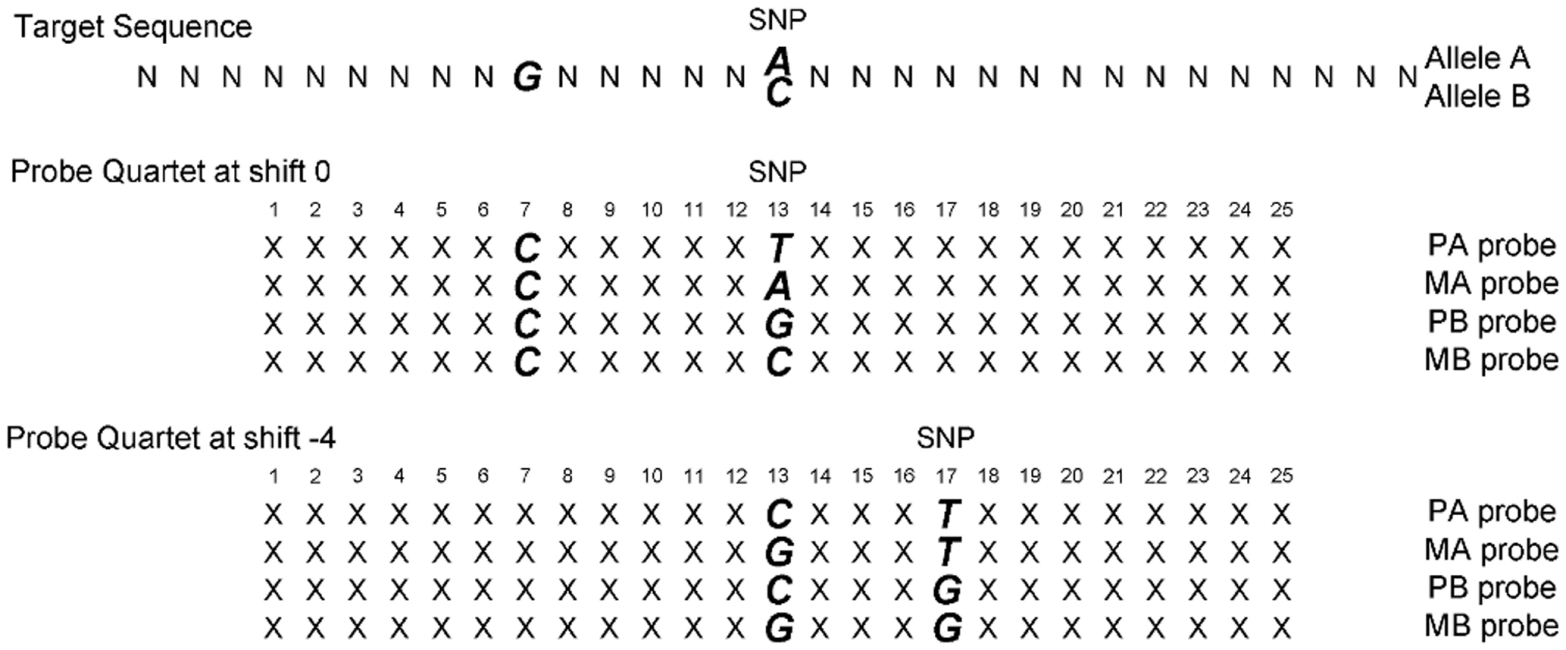
Twenty-five-mer oligonucleotides which are perfectly matched or mismatched to the target sequence with SNP allele A or B.

Affymetrix SNP arrays use multiple probe_sets to capture the property of each SNP. Here, we use Affymetrix Mapping 100 K arrays as an example to illustrate the design. In a 100 K array, ten quartets are used to interrogate a single dimorphic SNP site with alleles commonly denoted as *A* and *B*. Each quartet consists of 4 types of probes that are 25-mer in length, either perfectly matched to the target or mismatched at a particular SNP site for each allele: perfect match *A*, mismatch *A*, perfect match *B* and mismatch *B*, denoted respectively PA, MA, PB and MB for short. The quartets have different shifts (*k*) of the nucleotide (*k* may take the values −4, −3, −2, −1, 0, 1, 2, 3, 4) from the center of the probe sequence (*k*  =  0 at position 13 of the 25-mer), see Figure 1 for detailed illustration (Figure 1 was adapted from Figure 1A of [26]). The Affymetrix Mapping 500 K SNP Array has a similar design with 100 K arrays, but only 6 quartets are used to interrogate each SNP instead of 10.

In order to account for potential spatial effect on an array, the probe_sets of each SNP are usually distributed evenly over the array. The intensities for these probe_sets are highly correlated and inherently determined by SNP alleles. Unless the majority of array is defective, most of the probe_sets will not be affected. Therefore, it is possible to impute the affected intensities with those that are unaffected.

### PICR model for copy number estimation and genotype calling

In the past few years, a number of genotyping approaches, such as BRLMM and CRLMM, have been proposed for Affymetric high density SNP arrays [20], [21]. Recently, a single-array approach was proposed for copy number estimation and SNP genotype calling, referred to as Probe Intensity Composite Representation (PICR) [27]. It was shown previously that PICR attained higher genotyping accuracies than a commonly adopted approach, CRLMM. The PICR approach is based on single array training, and estimates copy number abundance on a single SNP basis. It addresses the cross-hybridization through the binding free energies and affinities between probes and targets. The free energy is calculated based on either perfect match or mismatch binding. It should be noted that a target sequence with allele *A* could hybridize to PA probe through perfect match binding and hybridize to PB, MA or MB probes through mismatch binding. Importantly, the hybridization to mismatch probes can have two mismatch nucleotides when shift *k ≠* 0 (Figure 1). Given the quartet (PA, MA, PB, MB) with shift *k*, the corresponding binding free energy is calculated with a positional-dependent nearest neighbor (PDNN) model [28], [29].

Following the same notation with Wan *et al.*, we first briefly introduce PICR model, the detail of which can be found elsewhere [27]. A target sequence TA with allele *A* could hybridize to PA probe through perfect match binding, the binding free energy of which is :
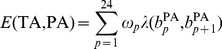
(1)where 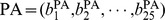 is the 25-mer PA probe, 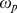 is a weight factor that depends on the position of consecutive bases along the probe, and 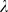 represents stacking energy depending on nearest neighbor along the probe.

Further, a target sequence TA with allele *A* could hybridize to MA, PB, MB (*k* = 0) probes through mismatch binding with one unique mismatch. For example, the binding free energy between TA and MA is
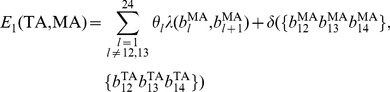
(2)


where 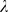 is the same stacking energy in Equation (1) and 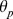 is a position weight factor for mismatch. 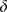
is the corresponding stacking energy for the triplets at mismatch position.

Finally, a target sequence TA with allele *A* could hybridize to MB probes through mismatch binding with two mismatches when shift *k ≠* 0. The mismatch would occur at both the center and shift *k* of the probe. The binding free energy is: 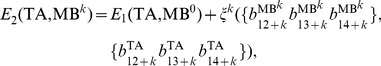
(3)


The term 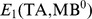 in Equation (3) is the binding free energy if there is only one unique mismatch at the center, and the term 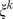 is the binding free energy of the triplets at the second mismatch.

Eventually, a linear regression model was used to model the probe intensities of quartets: 
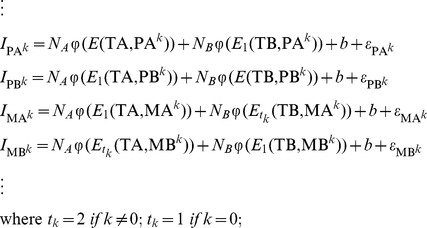
(4)where 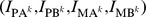 is the probe intensities of a quartet with shift *k*. *E* is the binding free energy between the probe and the target. 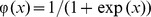 gives the corresponding binding affinity in the form of Langmuir adsorption [28], [30]. The coefficients 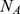 and 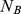 are copy number abundance of target sequences with allele *A* and allele *B*, respectively. The model intercept *b* represents the baseline intensity. The error terms 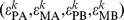 are independent measurement errors of intensities, each following a normal distribution with mean 0 and a constant variance. All the parameters are trained by a single HapMap sample, and are entirely determined by probe sequences. Empirically, we found the parameters were robust across different samples and different platforms, providing unbiased estimation of copy number abundance [27]. For a randomly selected HapMap sample, the linear model had a median R-square value of 0.764.

### Mixed effect model for multiple SNPs

The PICR model is a single-SNP, single-array approach, which was shown to have greater genotyping accuracies than a commonly used approach, CRLMM [27]. However, it also has a few limitations. First, the PICR model assumes the probe_sets of each SNP are mutually independent, which may not always be valid. Second, the PICR model utilized a linear regression framework with a very limited sample size (40 for 100 K array, 24 for 500 K array). It is less robust with outliers, such as probe intensities from defect areas. In this study, we extended the PICR model to a multi-SNP setting with a mixed effect model. In the extended model, we take into account the correlation among probe_sets for each SNP. Further, by adopting a mixed effect model, we borrow strength from multiple SNPs and improve its robustness to outliers. Finally, we are able to impute the intensities from defect areas by using a large number of SNPs that are not affected.

Assuming we have a SNP array with a large number of *N* SNPs, each has *M* probe_sets (*M* = 40 for 100 K SNP arrays). Each probe_set has binding affinities 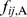 and 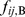 for target sequences TA and TB, respectively. The binding affinities can be calculated based on the equations described in Section 2.2. For example, if the *j*-th probe is a PA probe with shift *k*, then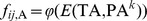, 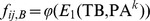.We model the intensity values for the *j*-th probe_set of the *i*-th SNP with a mixed effect model:



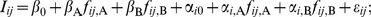
where 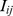 is the intensity for the *j*-th probe of SNP *i* ; *i* = 1, 2 ……*N*; *j* = 1, 2, ……40; and 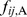, 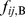 are the binding affinities of the *j*-th probe of SNP *i* with respect to two allele *A* and *B*, respectively. Here, 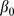, 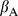, 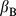 are the fixed effect for the baseline intensity, copy number abundance for allele *A* and copy number abundance for allele *B*, respectively; 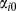,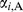,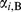 are the corresponding random effect subjected to SNP-wise variability with 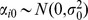, 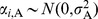, and 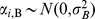; 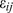 is a random measurement error with 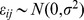. By using the above mixed effect model, the intensity values are assumed to be independent across different SNPs, but correlated within the same SNP. The fixed effect 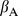, 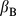 has an interpretation as the average copy number abundance of the *N* SNPs of interest. Further, 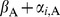,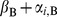 has an interpretation as the copy number abundance for the *i*-th SNP. The random effect 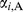,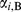 is a constant for all the available probes of SNP *i*, but varies across different SNPs, the variance of which are estimated by all probe intensities of *N* SNPs in the model.

Based on the above mixed effect model, an imputation procedure can be conducted as follows: 1) First, defect areas are identified by examining a CEL image or using existing image processing software, such as harshlight and BMR. The intensities from defect areas will be set as missing. 2) Second, for each affected SNP, a large number of unaffected SNPs (e.g. 100 SNPs) will be randomly selected to fit a mixed effect model. 3) The missing values can be imputed by the predicted values based on the mixed effect model.

## Results

**Figure 2:**
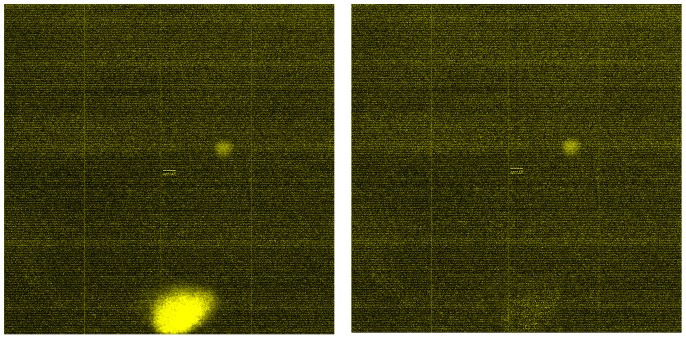
CEL images before and after imputation. Left: before, Right: after.

We considered 90 Affymetrix Mapping_100 K_Xba arrays from the HapMap study. D-ChIP was first used to examine the CEL image for each sample [31], [32], and 12 samples were identified with potential defect areas. Here, we only presented the CEL image for one sample (NA12812) as an example. The CEL images of the other 11 samples can be found in Figures S1, S2, S3, S4, S5, S6, S7, S8, S9, S10, and S11. The CEL image before and after imputation were given in Figure 2. Before imputation, a bright spot was identified at lower part of the image (Figure 2). Further examination indicated that over 20,000 SNPs were affected. However, for most of the SNPs, the number of affected probes is less than 10. We used a rectangle area to cover the bright spot and set the intensities from the defect area as missing. The data was then imputed according to the procedure described above. After imputation, the damaged area was recovered (Figure 2).

**Figure 3:**
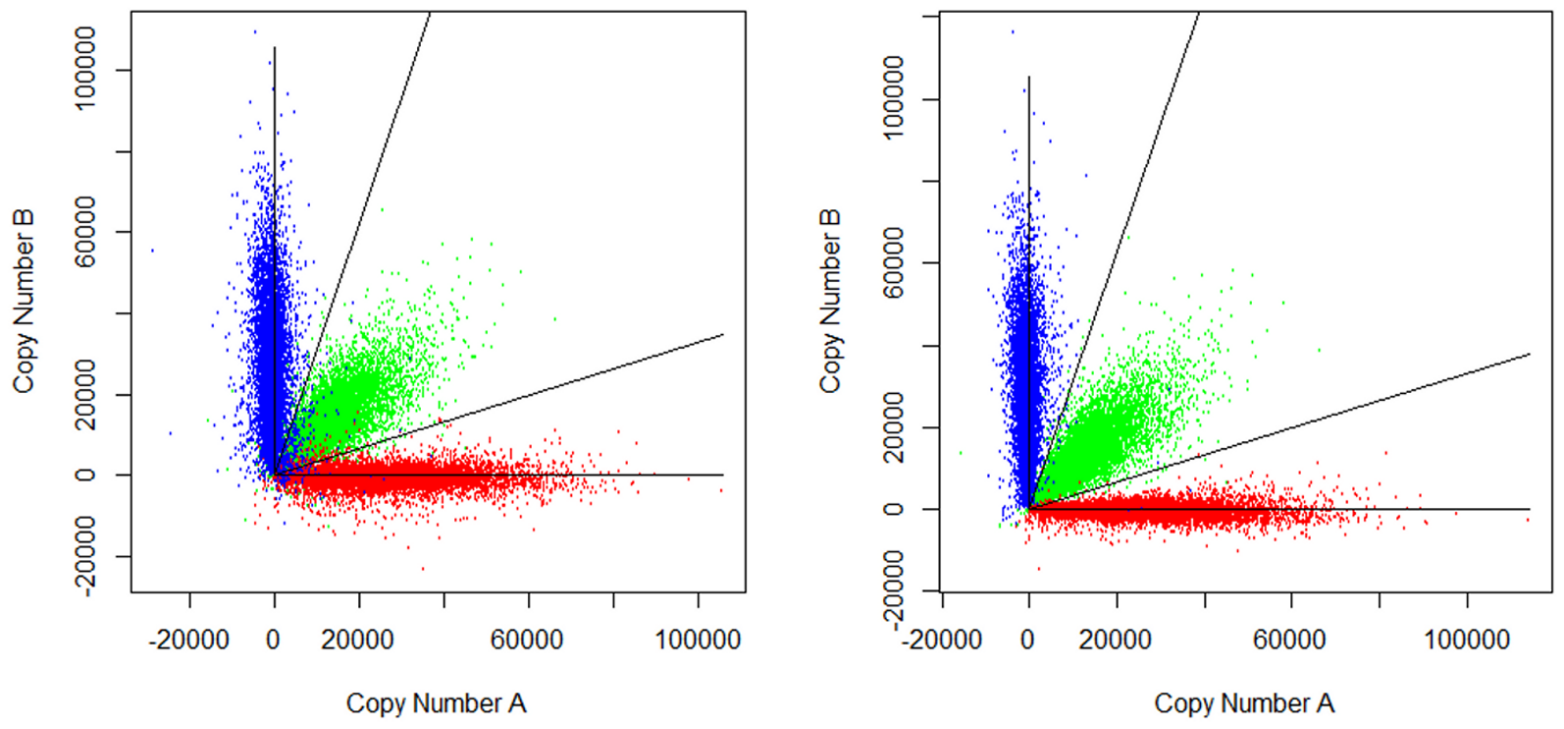
Allelic copy number abundance before and after imputation. Left: before, Right: after; Red: genotype AA, Blue: genotype BB, Green: genotype AB

In order to examine the quality of imputation, PICR was applied to conduct genotyping for both the original CEL and the imputed CEL. Figure 3 showed the estimation of allelic copy number abundance before and after imputation. PICR model conducted genotype calling by clustering all SNPs into three groups, corresponding to three possible genotypes, AA, AB and BB. The genotyping error rate was calculated as the discordant rate between PICR-genotype-calls and HapMap gold standards. Before imputation, applying PICR yielded a genotyping error rate of 2.54%, while the average genotyping error rate was 0.4% among all 90 samples. After imputation, the genotyping error rate was reduced by over 5 folds, to a normal level of 0.44%. The genotyping error rates for all 12 problematic samples were listed in Table 1. The results showed that the error rates were reduced for all 12 samples, especially for those with an error rate greater than 1% before imputation. Further examination showed the estimation of allelic copy number abundance was substantially improved after imputation.

**Table 1:** Genotyping error before and after imputation.

Sample ID	# of SNP affected	Ave. # of probe affected/SNP	Error Rate Before Imputation	Error Rate After Imputation
NA12812	20075	5.67	2.54%	0.45%
NA10835	20925	3.76	1.56%	0.32%
NA12239	12758	3.34	1.05%	0.26%
NA12144	7330	3.22	0.95%	0.24%
NA12005	4404	3.01	0.83%	0.65%
NA12056	7490	3.28	0.87%	0.85%
NA12146	7657	3.16	0.73%	0.25%
NA12155	9394	3.18	0.71%	0.71%
NA07056	2026	2.94	0.70%	0.54%
NA12236	8071	3.19	0.66%	0.65%
NA12813	4788	3.15	0.61%	0.25%
NA10863	5050	3.07	0.56%	0.55%

According to the number (proportion) of affected probes, we further classified all SNPs into four groups, including 0 affected probe, 1–4 affected probes (<10%), 5–8 affected probes (10%–20%) and >8 affected probes (>20%). The result was summarized in Table 2. Most of the affected SNPs had 1–4 affected probes (<10%). Before imputation, the error rates increased dramatically with the number of affected probes, from 3.3% (1–4 affected probes) to 9.19% (>8 affected probes). After imputation, the error rates were significantly reduced for all groups. While the error rate was still considerably lower for SNPs in <10% group, no significant difference was found between 10–20% group and >20% group. The imputation appeared to be effective for all groups, and was able to reduce the error rate by as high as 13 folds. For Affymetrix 500 K SNP arrays, we expected the number of affected probes to be proportional. A similar defect area would affect a lot more SNPs, but less number of probes for each SNP.

**Table 2:** Genotyping error rates stratified by number of affected probes.

# of affected probes	0	1–4 (<10%)	5–8 (10%–20%)	>8 (>20%)
# of SNPs	415417 (84.5%)	65458 (13.3%)	8313 (1.7%)	2688 (0.5%)
# of errors before imputation	1871	2165	504	247
Error rates before imputation	0.45%	3.33%	6.06%	9.19%
# of errors after imputation	1871	379	74	19
Error rates after imputation	0.45%	0.58%	0.89%	0.71%
Fold Change of Error rates	1	5.7	6.8	13

## Discussion

In this study, we have proposed a multi-SNP approach for imputing the intensities from defect areas on Affymetrix SNP arrays. The approach can be viewed as an extension of previously proposed PICR model. Similar to PICR, it is a single-array approach, which does not require any biological replicate or between-array normalization. It would be especially helpful for small studies with limited sample sizes. Further, the results have showed that this approach is effective to impute intensities from defect areas. The genotyping error rates were significantly reduced.

In this article, we have focused on Affymetrix SNP arrays, because the performance can be easily examined by genotyping error rates. However, our approach models the fundamental mechanism of physical binding between DNA nucleotides. It can potentially be extended to other oligonucleotide microarrays with a similar design. For example, the gene expression arrays have similar PM/MM probe_sets, with the number of pairs ranging from 11–16. To extend the proposed approach to gene expression microarrays, the binding free energy will need to be re-calculated. Zhang *et al.* have investigated the sequence-specific binding and non-specific binding for gene expression data, and provided estimation for the corresponding binding free energy [28], [29]. Further adjustment will also be necessary to accommodate the diverse number of probe_sets for each gene/transcript, and the competing hybridization process of DNA sequences from various experimental conditions.

In our study, we also found that the genotyping error rates for a few arrays remained similar after imputation, e.g. NA10853. One reason was that the defect area was relatively small and only a small number of SNPs were affected. Given the large number of total SNPs on the array, it did not have a great impact on the overall genotype calling. Another possible reason is that the genotyping errors can be caused by multiple sources. For those arrays, there might be other possible factors affecting genotyping accuracies.

One remaining problem of the proposed approach is to efficiently identify the boundary for defect areas. The defect areas on an array may have irregular shapes. In this article, we have used a rectangle area to cover the defect area for imputation. However, these rectangles were slightly larger than the bright spots and some unaffected intensities are set to missing as well. It will be helpful to identify the boundary of defect areas effectively and accurately. In addition, other studies have shown that some outliers can not be easily identified by checking CEL images. Several computational algorithms have emerged as promising tools for detecting defect areas on a CEL image, such as ‘harshlight’ and ‘BRB’. These available packages are potentially helpful to identify the outliers automatically.

## Supporting Information

Figure S1 **The CEL image before and after imputation for sample NA07056.**
(TIF)

Figure S2 **The CEL image before and after imputation for sample NA10835.**
(TIF)

Figure S3 **The CEL image before and after imputation for sample NA10863.**
(TIF)

Figure S4 **The CEL image before and after imputation for sample NA12005.**
(TIF)

Figure S5 **The CEL image before and after imputation for sample NA12056.**
(TIF)

Figure S6 **The CEL image before and after imputation for sample NA12144.**
(TIF)

Figure S7 **The CEL image before and after imputation for sample NA12146.**
(TIF)

Figure S8 **The CEL image before and after imputation for sample NA12155.**
(TIF)

Figure S9 **The CEL image before and after imputation for sample NA12236.**
(TIF)

Figure S10 **The CEL image before and after imputation for sample NA12239.**
(TIF)

Figure S11 **The CEL image before and after imputation for sample NA12813.**
(TIF)
