# Supplementary figures and images for: An Imputation Approach for Oligonucleotide Microarrays
Source: PLoS One. 2013 Mar 7;8(3):e58677. doi: 10.1371/journal.pone.0058677 (PMC3591399; doi:10.1371/journal.pone.0058677)

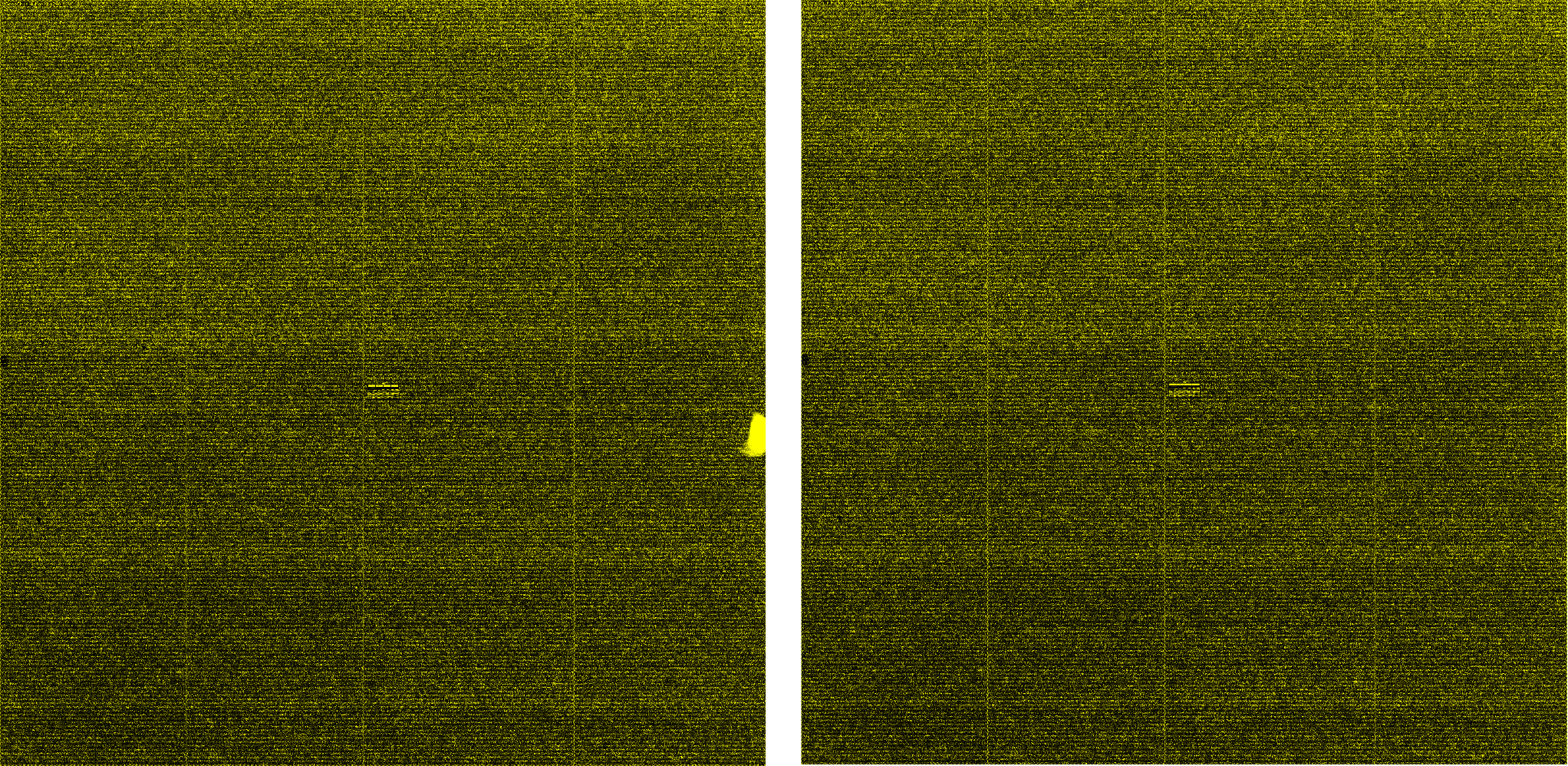

Supplement: Figure S1 — The CEL image before and after imputation for sample NA07056. (TIF) [file pone.0058677.s001.tif]

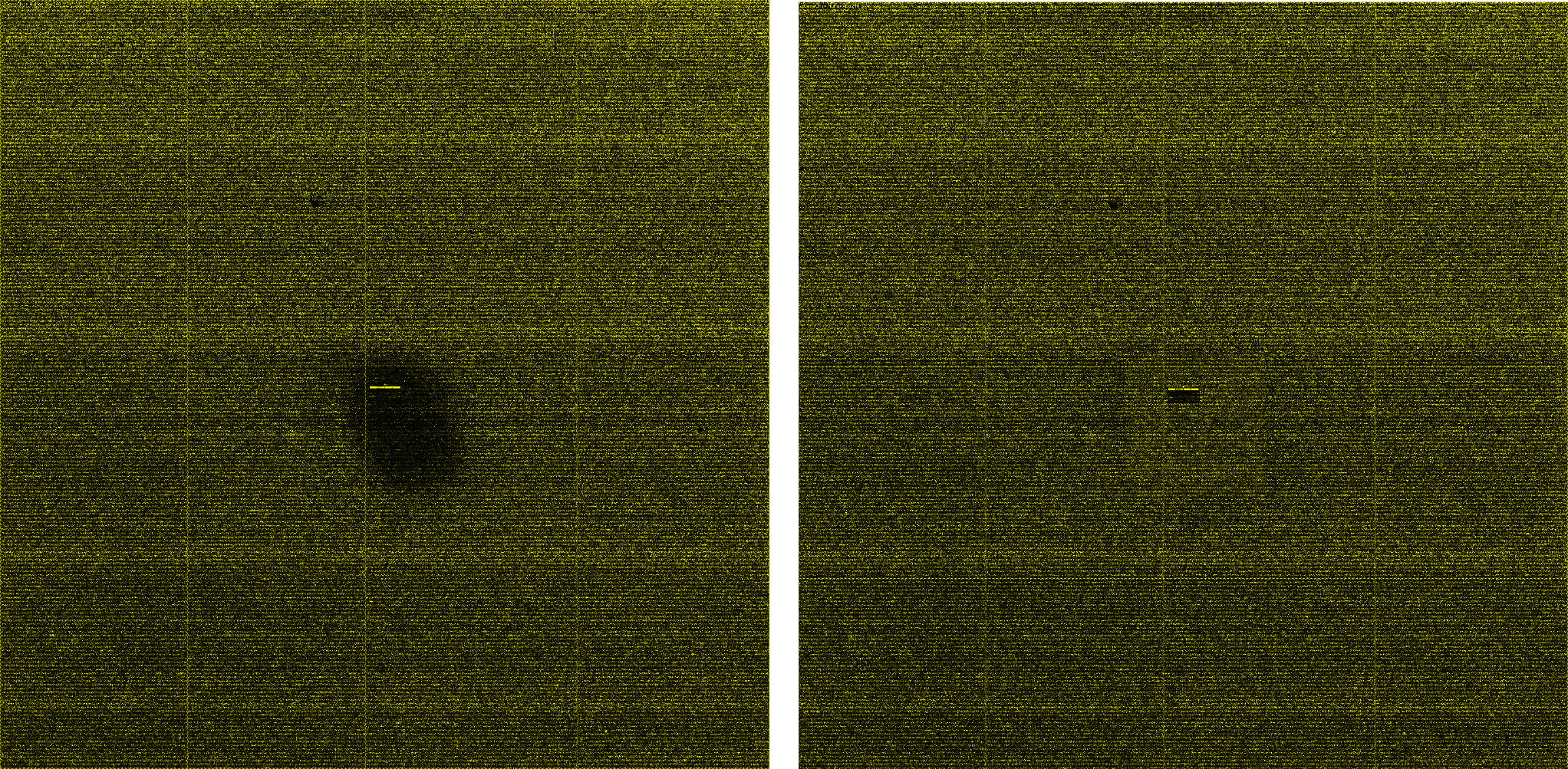

Supplement: Figure S2 — The CEL image before and after imputation for sample NA10835. (TIF) [file pone.0058677.s002.tif]

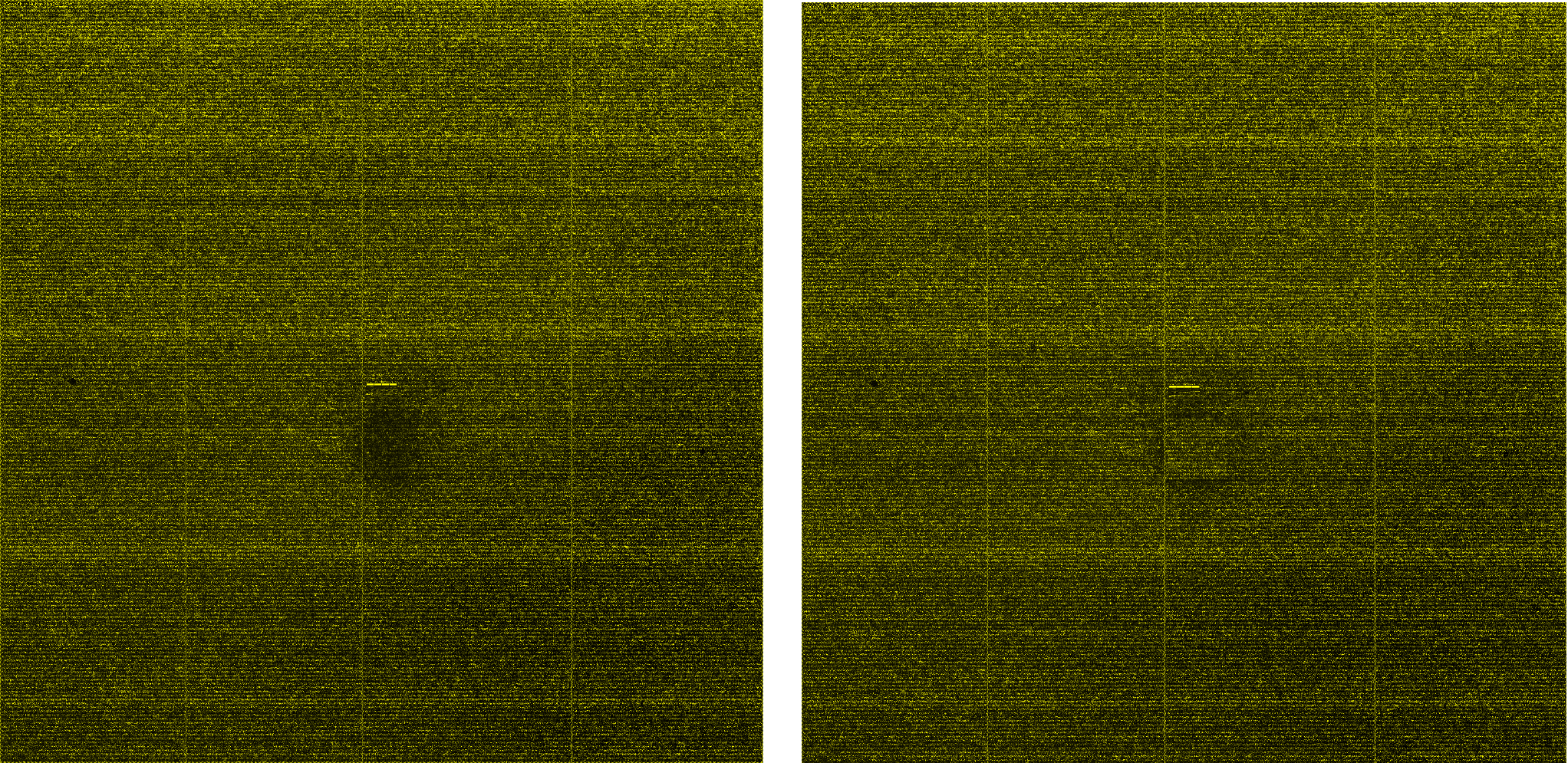

Supplement: Figure S3 — The CEL image before and after imputation for sample NA10863. (TIF) [file pone.0058677.s003.tif]

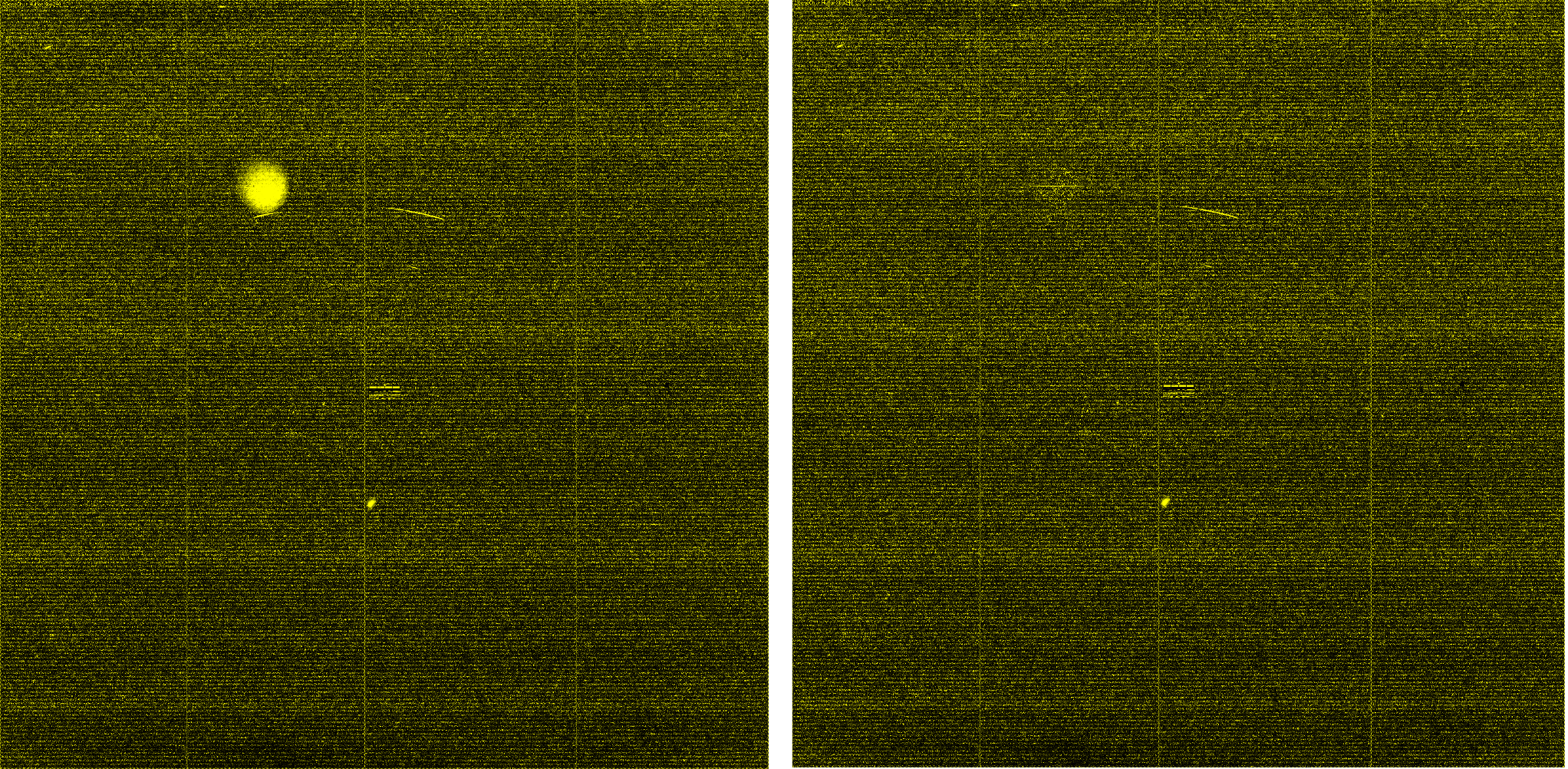

Supplement: Figure S4 — The CEL image before and after imputation for sample NA12005. (TIF) [file pone.0058677.s004.tif]

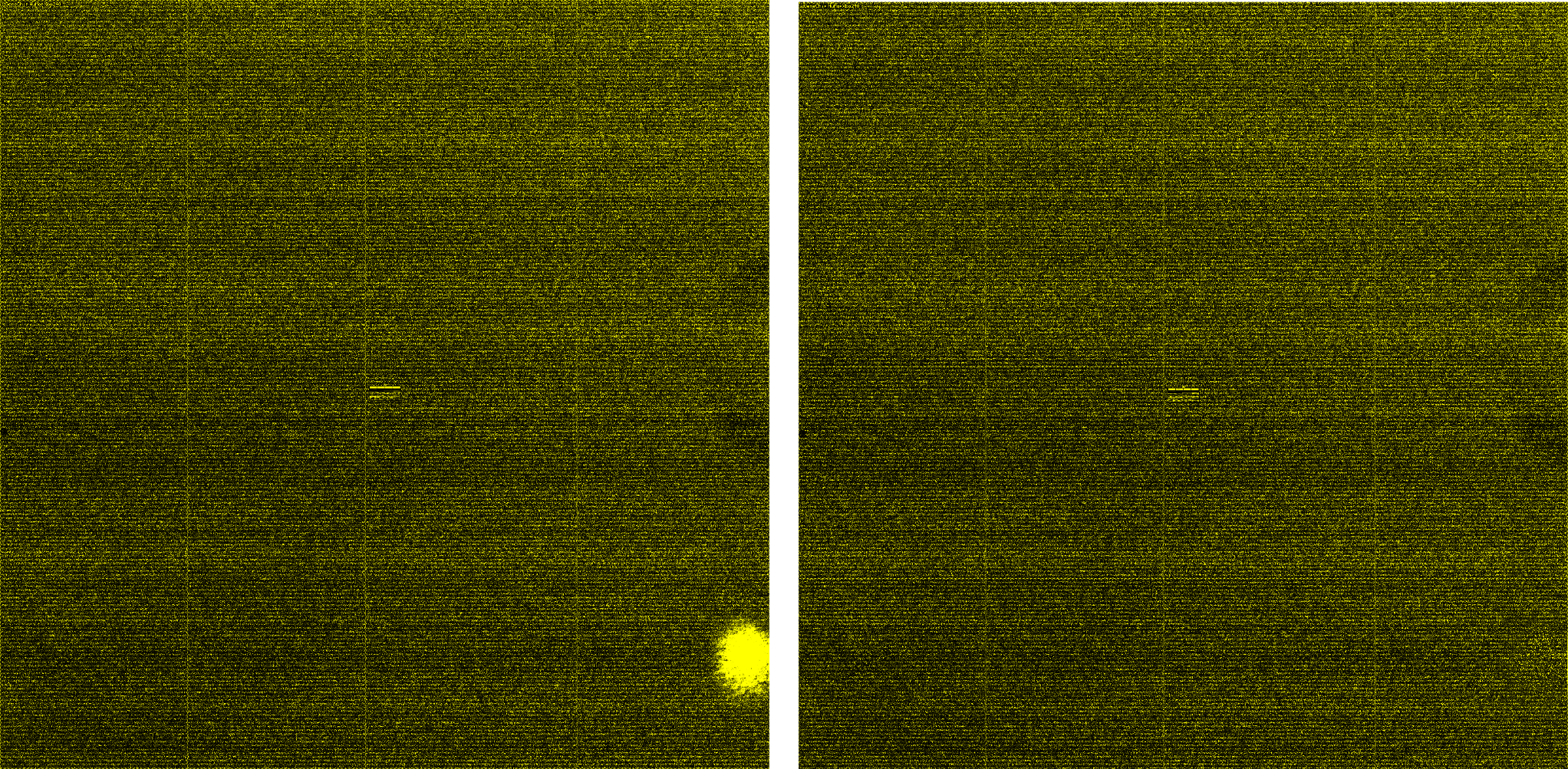

Supplement: Figure S5 — The CEL image before and after imputation for sample NA12056. (TIF) [file pone.0058677.s005.tif]

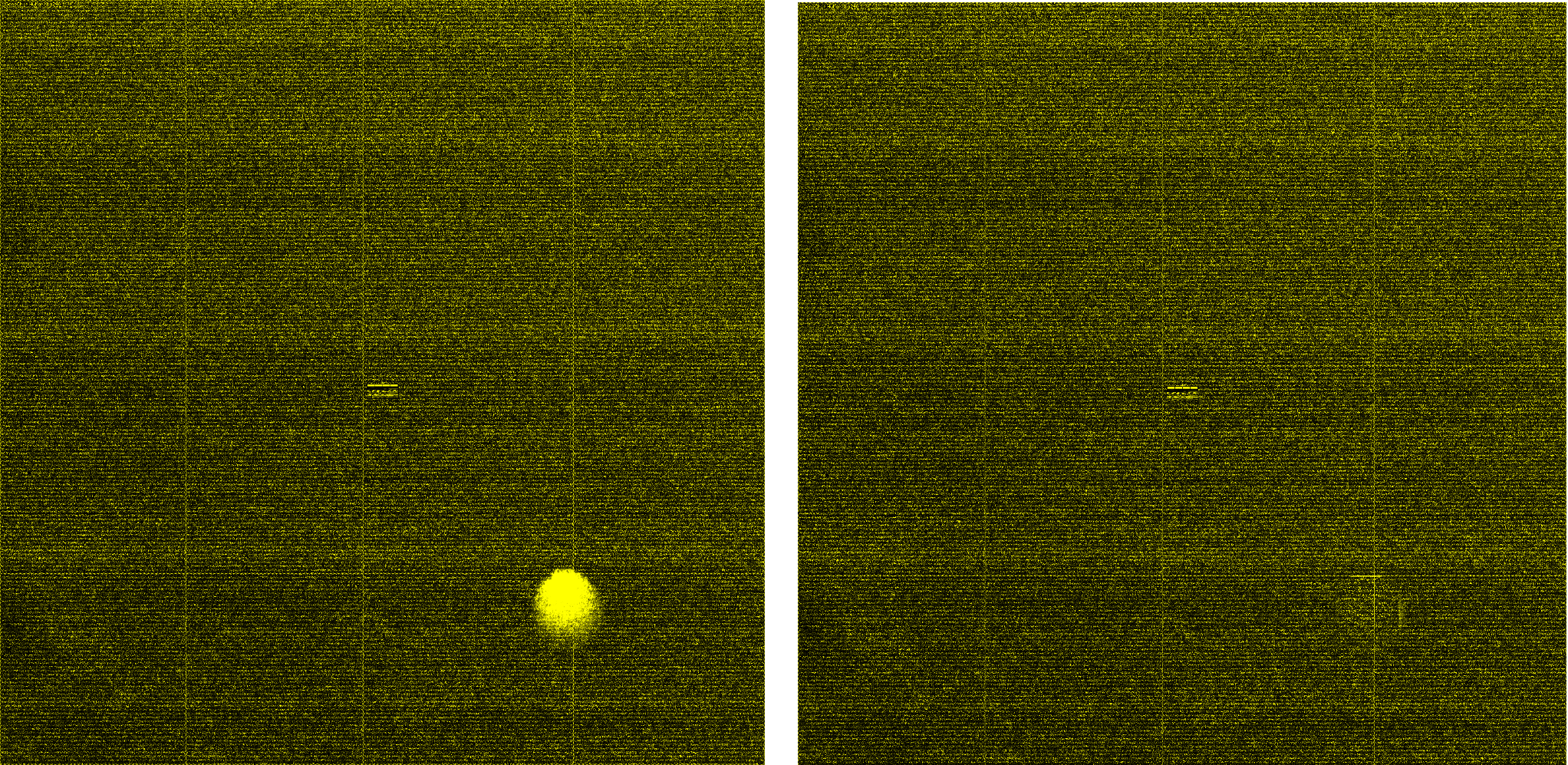

Supplement: Figure S6 — The CEL image before and after imputation for sample NA12144. (TIF) [file pone.0058677.s006.tif]

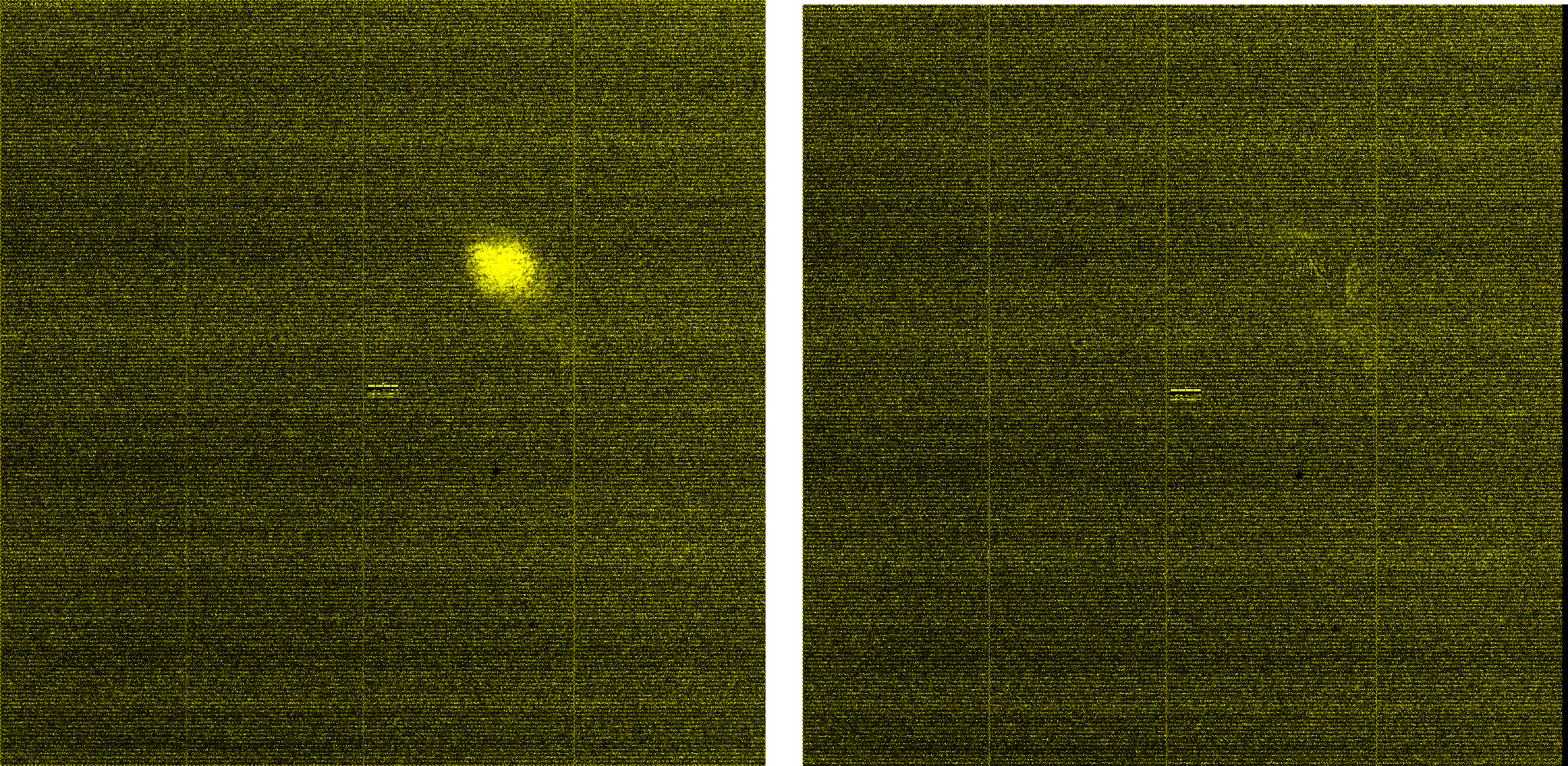

Supplement: Figure S7 — The CEL image before and after imputation for sample NA12146. (TIF) [file pone.0058677.s007.tif]

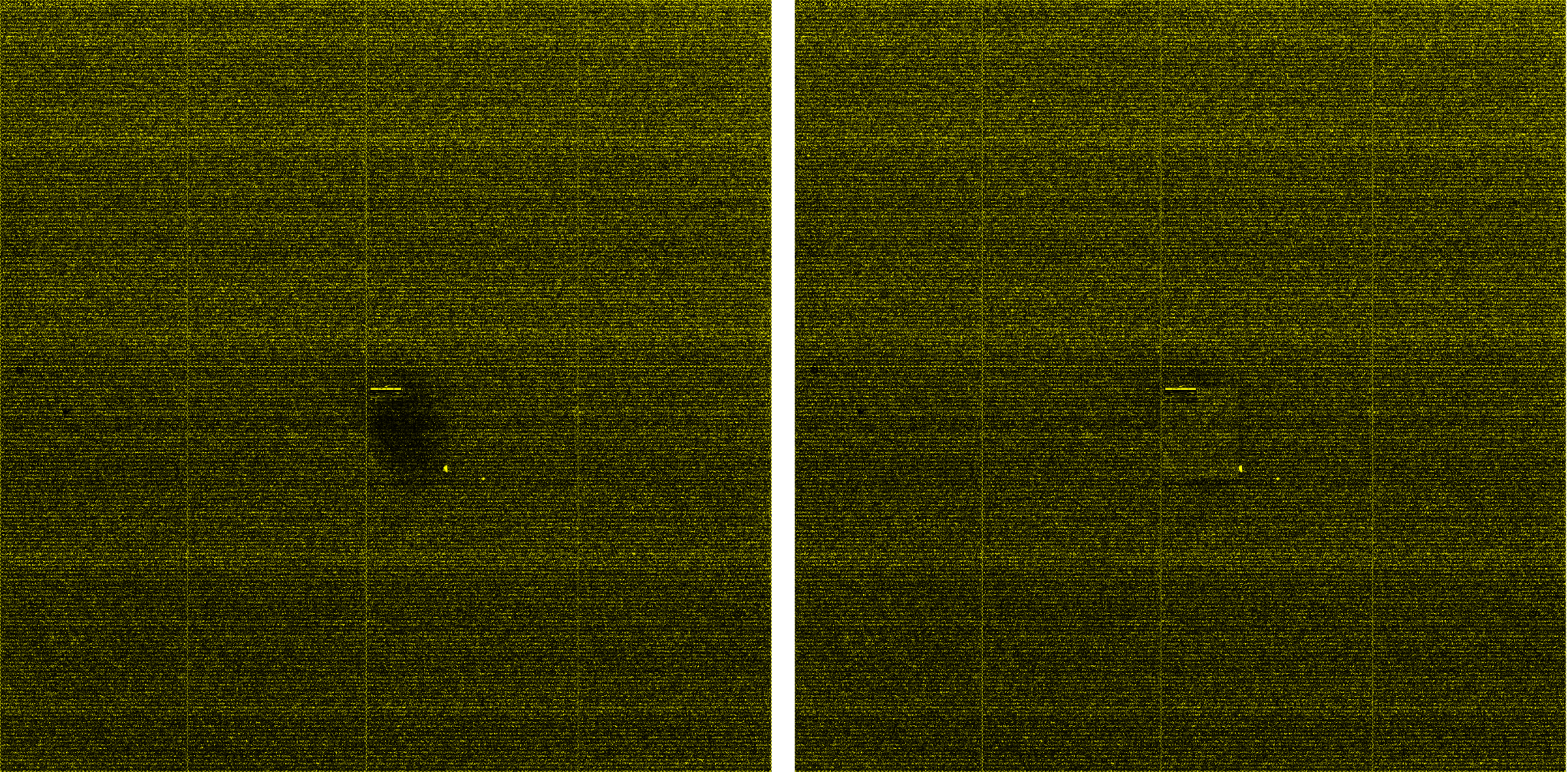

Supplement: Figure S8 — The CEL image before and after imputation for sample NA12155. (TIF) [file pone.0058677.s008.tif]

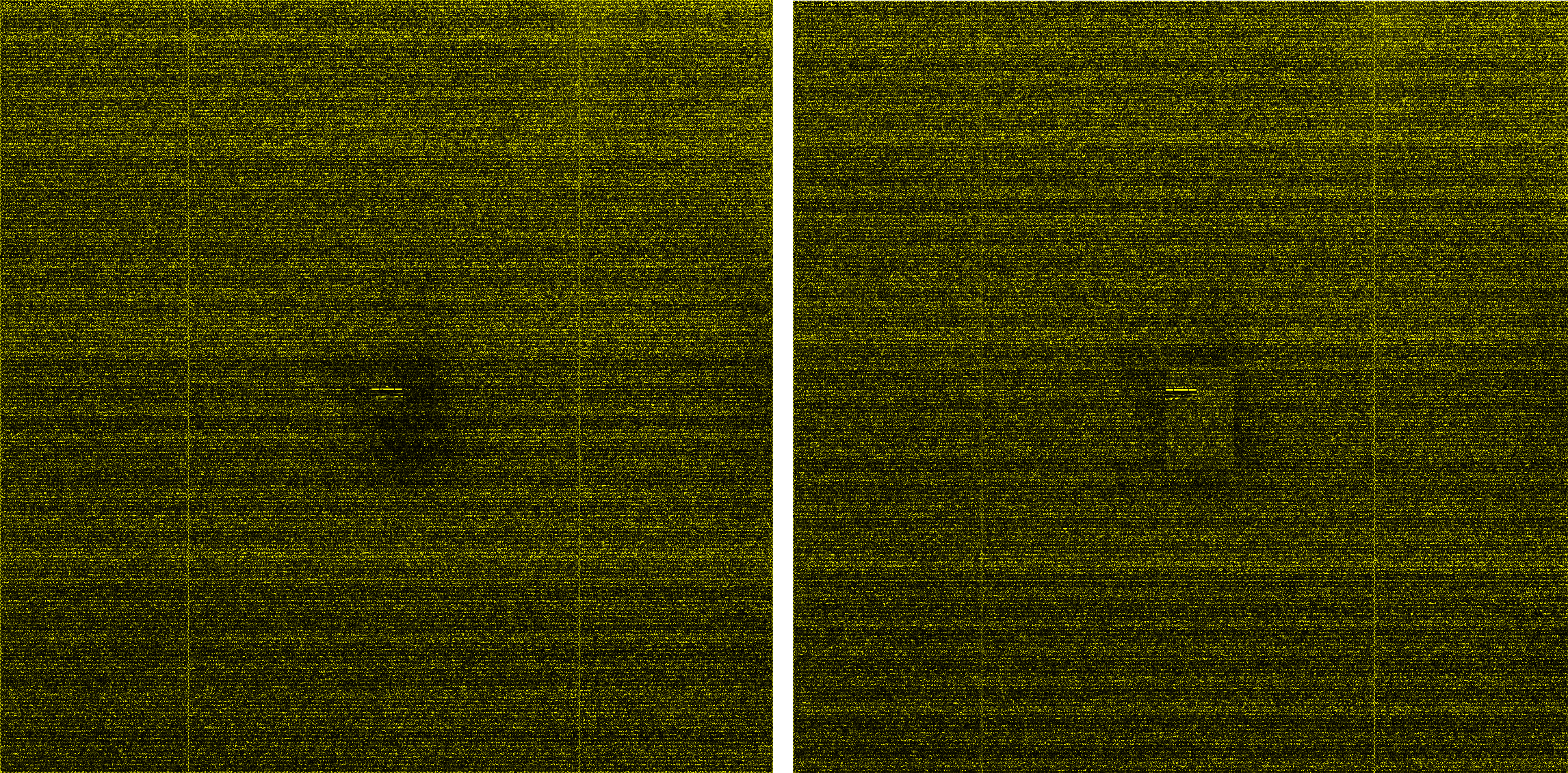

Supplement: Figure S9 — The CEL image before and after imputation for sample NA12236. (TIF) [file pone.0058677.s009.tif]

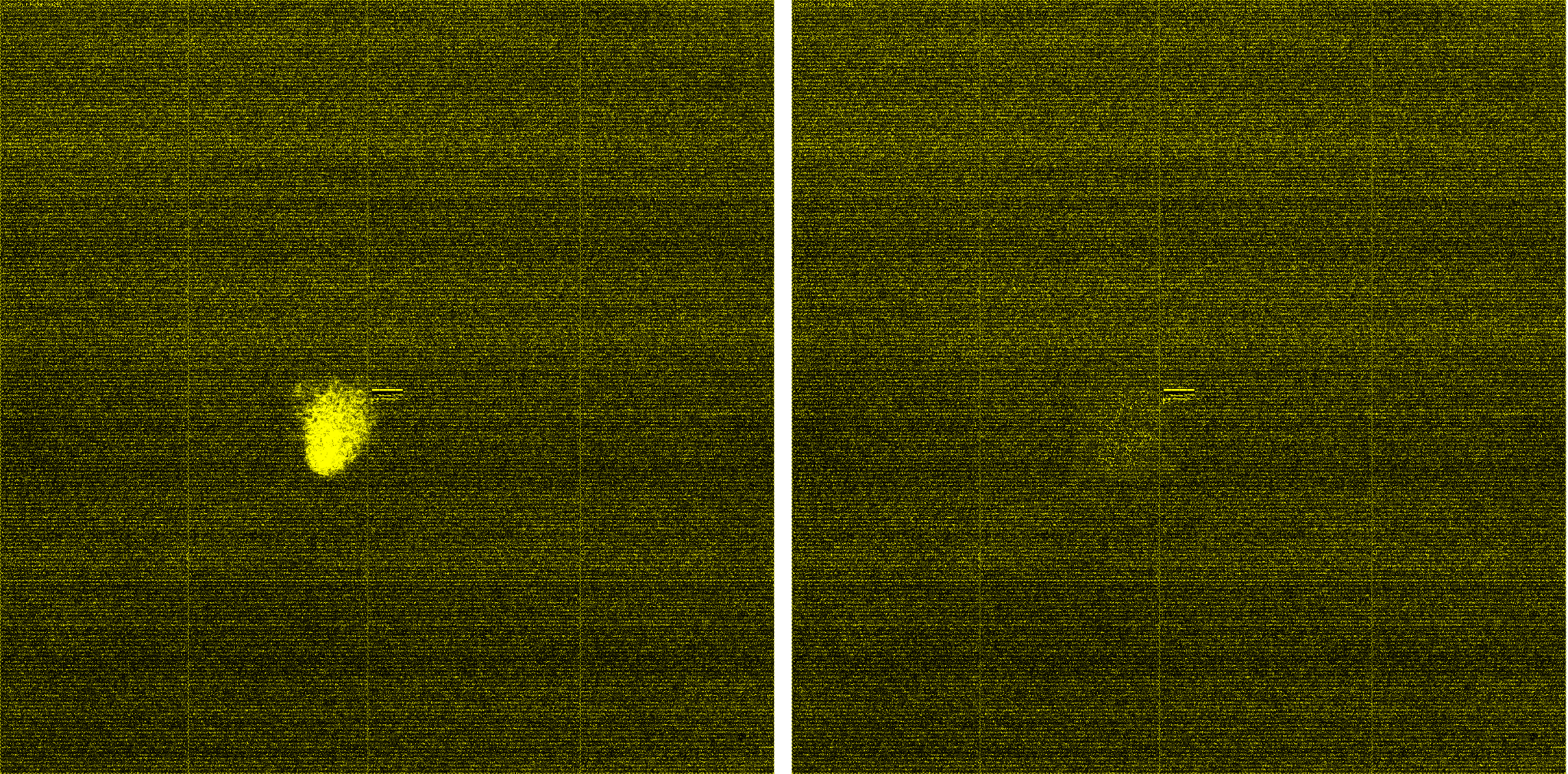

Supplement: Figure S10 — The CEL image before and after imputation for sample NA12239. (TIF) [file pone.0058677.s010.tif]

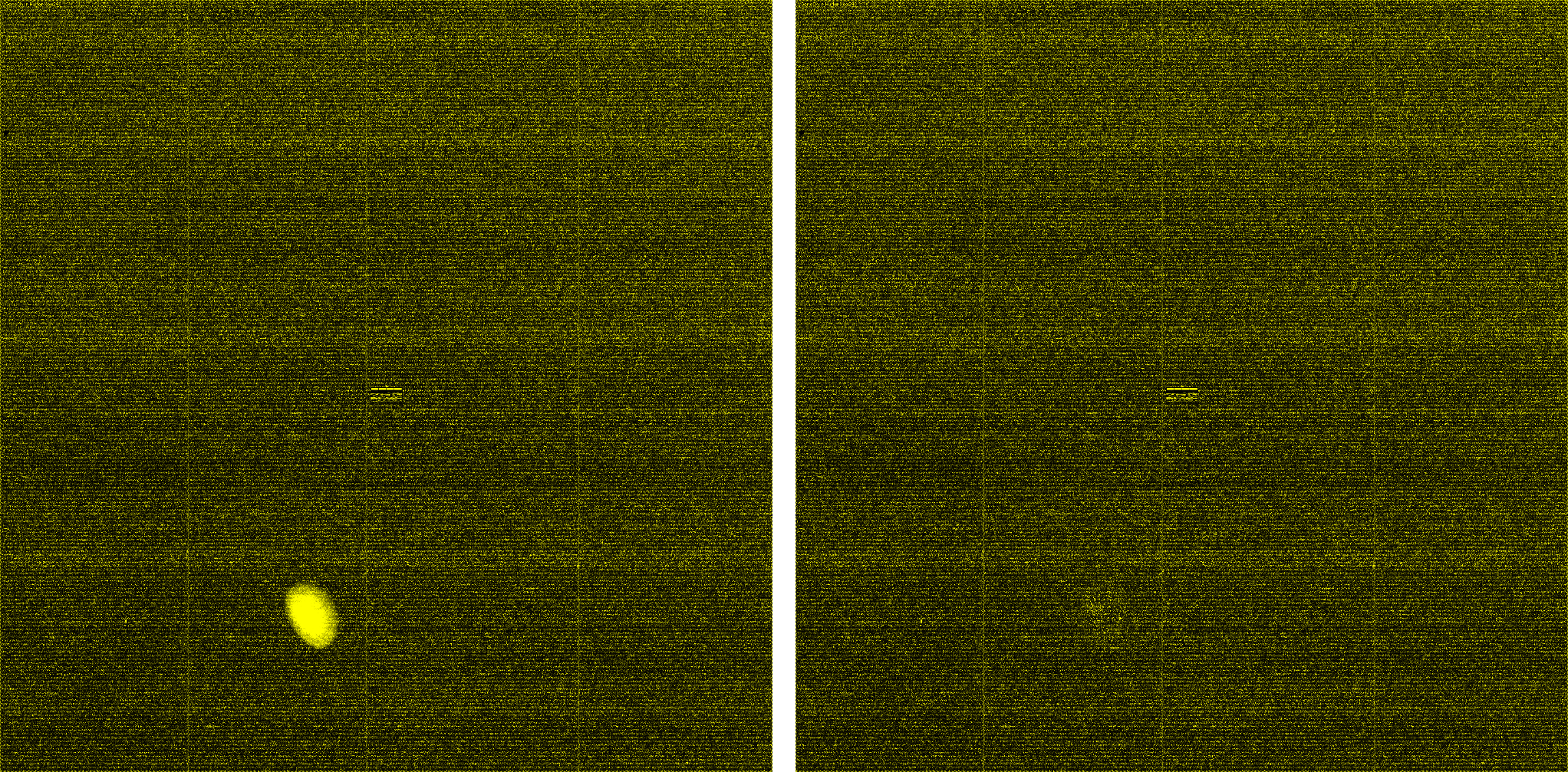

Supplement: Figure S11 — The CEL image before and after imputation for sample NA12813. (TIF) [file pone.0058677.s011.tif]
